# Polarization Multiplexing Terahertz Quasicrystal Meta-Platform

**DOI:** 10.3390/ma19153162

**Published:** 2026-07-23

**Authors:** Zhanfan Li, Meng Liu, Shuo Guan, Keke Cheng, Jiaxing Shi, Xianrui Jiang, Haiping Wu, Hongyue Gao, Dehua Li, Wei Yan, Huiyun Zhang, Yuping Zhang

**Affiliations:** Qingdao Key Laboratory of Terahertz Science, Technology and Applications, College of Electronic and Information Engineering, Shandong University of Science and Technology, Qingdao 266590, China

**Keywords:** terahertz, all-dielectric metasurface, quasicrystal, multifunctional meta-device, polarization multiplexing

## Abstract

Multidimensional metasurfaces provide a promising platform for terahertz (THz) multifunctional devices used in communication, imaging, and sensing. However, many THz multifunctional devices still rely on metallic or periodic metasurfaces, which may suffer from ohmic loss, unwanted diffraction, channel crosstalk, and energy leakage. To address these limitations, we propose an all-dielectric THz metasurface based on a five-fold rotationally symmetric quasicrystalline aperiodic tiling and verify its performance through full-wave electromagnetic simulations. High-resistivity silicon rectangular pillars are used as anisotropic propagation-phase meta-atoms, enabling independent wavefront encoding for two orthogonal linear polarizations within a single aperture. By mapping the *x*- and *y*-polarized phase profiles onto the quasicrystalline lattice, the proposed device realizes polarization-multiplexed bifocal focusing with controllable focal positions. Simulation results show high focusing efficiency, low polarization crosstalk, broadband focusing performance, and robustness under oblique incidence. This work provides a compact all-dielectric route for multifunctional THz wavefront control based on polarization multiplexing and quasicrystalline metasurface design.

## 1. Introduction 

In recent years, metasurfaces composed of subwavelength artificial microstructures have become important platforms for optical and photonic wavefront control because they can introduce abrupt spatial phase discontinuities [1]. The concept of designer flat optics further established metasurfaces as compact planar optical elements [2]. Representative demonstrations include gradient metasurfaces for controlling propagating and surface waves [3], metasurface holography [4,5], dielectric gradient optical elements [6], spin-to-orbital angular momentum conversion [7], complete phase and polarization control with dielectric metasurfaces [8], diffraction-limited metalenses [9], and recent THz metalens engineering [10]. However, many metasurface designs still rely on periodic lattices. Although such lattices are convenient for modeling and fabrication, their translational symmetry can limit the design freedom required for broadband and multifunctional wavefront control [11,12].

Quasicrystals provide an alternative structural platform because they possess long-range order without translational periodicity. Early theoretical work introduced quasicrystals as a new class of ordered structures [13], and their geometric features were later systematically summarized [14]. Experimentally, Shechtman et al. observed metallic structures with long-range orientational order and no translational symmetry, including fivefold rotational symmetry [15]. This concept was subsequently extended to photonics: Cheng et al. investigated defect and transmission properties in two-dimensional quasiperiodic photonic band-gap systems [16], and Zoorob et al. demonstrated complete photonic band gaps in 12-fold symmetric quasicrystals [17]. More recently, quasicrystal metasurfaces have been introduced as a new design dimension for THz metalenses [18]. Related studies of aperiodic and optimized photonic structures also show that non-conventional spatial arrangements can provide useful photonic band-gap or wavefront-control properties [19,20].

Introducing quasicrystalline arrangements into two-dimensional metasurfaces may therefore transfer the ordered but non-periodic features of quasicrystals to planar wavefront-control devices. Structured optical fields have been used for high-capacity free-space data transmission based on orbital angular momentum multiplexing [21], while all-dielectric metasurfaces have enabled nonlinear wavefront control [22]. Metamaterials have also been proposed for wave-based mathematical operations [23]. In the THz regime, spin-enabled quasicrystal metasurfaces have recently been used for orbital angular momentum engineering, demonstrating the potential of quasicrystalline lattices for complex wavefront encoding [24,25]. In parallel, advances in metalenses [26], high-quality-factor phase-gradient metasurfaces [27], gradient metasurface theory [28], and nonlinear metasurface materials and fabrication [29] have broadened the functional scope of planar photonic devices. Nevertheless, the integration of quasicrystalline spatial arrangements with polarization multiplexing for efficient multifunctional THz devices remains insufficiently explored.

Modulated by the metasurface and focused to different focal points in the first and third quadrants, respectively, achieving polarization-multiplexed optical focusing functionality. The illustration shows a local magnified structure of the metasurface. On the right side, the two-dimensional cross-sectional intensity distribution at the focal points of the two orthogonal polarization states is presented (in the *y–z* plane and *x–y* plane, respectively).

**Figure 1:**
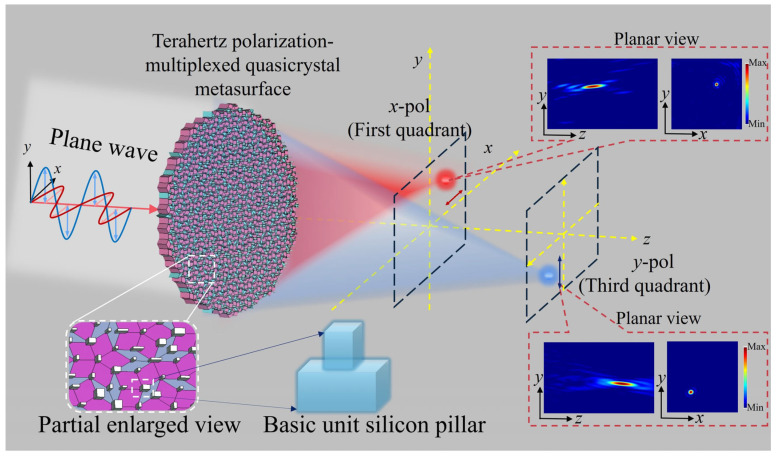
Schematic diagram of the working principle of a THz polarization multiplexing lens based on quasicrystalline metasurfaces. The incident *x*-polarized and *y*-polarized plane waves are.

In light of this, this paper proposes a fully dielectric THz metasurface based on a five-fold rotationally symmetric quasicrystalline aperiodic arrangement for polarization-multiplexed dual-focus control. As shown schematically in Figure 1, *x*- and *y*-polarized incident waves are independently focused to designed spatial positions within a single-layer device. Compared with previous metasurface strategies for polarization control [30] and all-dielectric focusing [31], the present design employs high-resistivity silicon rectangular pillars as anisotropic propagation-phase meta-atoms. High-contrast transmitarrays and dielectric Huygens surfaces have shown that dielectric resonators can provide efficient phase control [32,33], and recent THz all-dielectric metalenses have demonstrated multiple polarization-channel focusing [34]. Inspired by flat-lens wavefront engineering [35], polarization-selective metasurface control [36,37], inverse design [38], and efficient dielectric wavefront shaping [39], we encode the required *x*- and *y*-polarized focusing phase profiles onto the same quasicrystalline aperture. This work combines metasurface wavefront manipulation [40], phase-gradient and continuous metasurface concepts [41,42], and high-efficiency or tunable flat-optics strategies [43,44] to develop a polarization-multiplexed quasicrystalline THz metalens platform.

## 2. Meta-Atom Design and Database Construction

This paper aims to address this challenge by proposing and thoroughly discussing a THz dielectric metasurface based on a five-fold rotationally symmetric quasicrystal structure, which achieves dual-focus focusing functionality for linearly polarized light multiplexing for the first time in a single device. We have abandoned traditional periodic lattice points and adopted a Penrose tiling pattern, generated through six iterations, as a non-periodic arrangement template for the meta-atoms [45]. To independently control the wavefronts of two orthogonal linearly polarized states, this study selected anisotropic rectangular silicon pillars as the basic meta-atom unit. From the perspective of polarization optics, these units can be regarded as a linear birefringent wave plate, whose optical response can be described using Jones matrices [46,47]:

(1) $$J=R\left(-\theta \right)\left[\begin{array}{cc} e^{i\phi_{x}} & 0\\ 0 & e^{i\phi_{y}} \end{array}\right]R\left(\theta \right)$$

In this design, $\varphi_{x}\;$ and ${\;\varphi }_{y}$ represent the phase shifts introduced by the unit for *x*- and *y*-linearly polarized incident light, respectively, while  θ  denotes the orientation angle of the unit structure. We fix $\theta =0^{{}^{\circ}}$ so that the edges of the silicon pillars are aligned with the coordinate axes. Therefore, by independently controlling the phase shifts provided by each unit for ${(\varphi }_{x},\varphi_{y})$, we can construct arbitrary phase profiles $\Phi_{x}(x,y)$ and $\Phi_{y}(x,y)$ for the two polarization channels, thereby achieving the dual functionality of polarization multiplexing.

This study first establishes a comprehensive database of anisotropic silicon pillar structures. Subsequently, two independent focal phase planes corresponding to *x*- and *y*-linearly polarized light are precisely mapped to each position of a non-periodic quasicrystal array with perfect five-fold symmetry. This work not only demonstrates a novel dual-function focusing device but, more importantly, validates the significant value of the synergistic design paradigm of quasicrystal arrangement combined with polarization multiplexing in enhancing device performance. Through full-wave electromagnetic simulations, we prove that the quasicrystal dual-focus lens (DFL) exhibits high efficiency and low-crosstalk focusing characteristics near the design frequency point. The electromagnetic responses of the silicon pillar meta-atoms were simulated using CST Studio Suite 2020. 

Furthermore, due to its non-periodic structure’s intrinsic suppression advantages over periodic benchmark designs in terms of operational bandwidth and robustness against fabrication errors. This research provides a new design concept and implementation scheme for the development of the next generation of high-performance, highly integrated planar optical devices. To achieve the dual-focus function, it is necessary to first design the corresponding phase distributions $\Phi_{x}(x,y)$ and $\Phi_{y}(x,y)$ for *x*- and *y*-polarized light, respectively. For a lens with a focal length of f, if the desired focal point is located on the optical axis, the required phase distribution follows the spherical wave formula [48]:

(2) $$\Phi_{on-axis}\left(x,y\right)=\frac{2\pi }{\lambda }\left(f-\sqrt{x^{2}+y^{2}+f^{2}}\right)$$

where λ is the operating wavelength. When the desired focal point deviates from the optical axis and is located at the target point $\left(x_{0},y_{0}\right)$ on the focal plane, the required phase distribution is:

(3) $$\Phi_{\mathrm{off}-\mathrm{axis}\;}(x,y;x_{0},y_{0})=\frac{2\pi }{\lambda }\left(\begin{array}{c} \sqrt{f^{2}+x_{0}^{2}+y_{0}^{2}}-\\ \sqrt{{\left(x-x_{0}\right)}^{2}+{\left(y-y_{0}\right)}^{2}+f^{2}} \end{array}\right)$$

The proposed metasurface was designed using a target-phase-to-structure mapping strategy. First, the desired focal positions for *x*- and *y*-polarized incident THz waves were determined, and the corresponding phase profiles were calculated using Equations (2) and (3). High-resistivity silicon was selected because of its low absorption loss in the THz regime and compatibility with standard microfabrication. Rectangular silicon pillars were used as anisotropic meta-atoms because their two lateral dimensions can tune the effective optical paths of *x*- and *y*-polarized waves. By sweeping these dimensions, a phase and transmission database was established, from which 64 meta-atoms were selected to provide eight discrete phase levels for each polarization channel while maintaining relatively uniform transmission amplitudes.

The metasurface aperture was constructed using a fivefold Penrose-type quasicrystalline tiling generated through six iterations, with a rhombic edge length of 300 μm. Each lattice site was assigned a silicon pillar from the meta-atom database according to the required local phase pair for the *x*- and *y*-polarized channels. Thus, the two polarization-dependent phase profiles were encoded onto an ordered but non-periodic quasicrystalline aperture to form the final polarization-multiplexed THz metalens.

**Figure 2:**
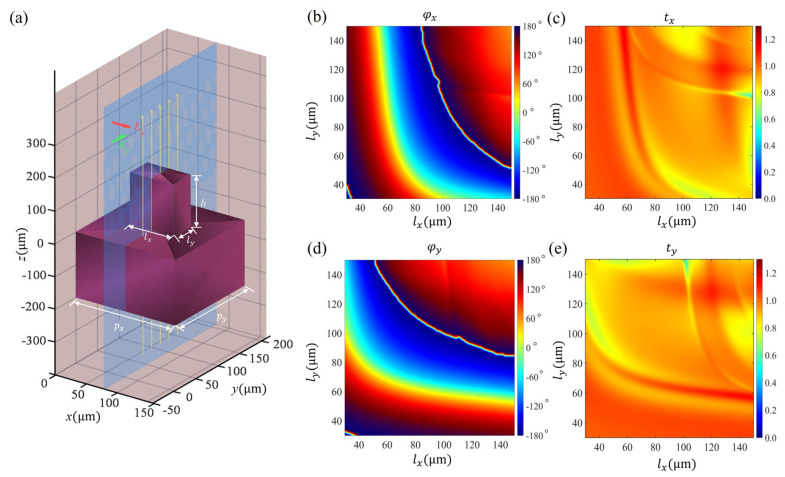
Simulation structural unit and database (**a**) Schematic diagram of the rectangular silicon pillar unit structure of polarization-dependent metasurface. (**b**,**c**) Phase $\varphi_{x}$ and transmission amplitude $t_{x}$ of the silicon pillar under *x*-polarized incidence at 0.9 THz. (**d**,**e**) Phase $\varphi_{y}$ and transmission amplitude $t_{y}$ under *y*-polarized incidence at 0.9 THz.

To construct a structural database for wavefront design, we conducted systematic electromagnetic simulations of silicon pillar units. In the simulations, as shown in Figure 2a, the side lengths $l_{x}$ and $l_{y}$ of the rectangular silicon pillars varied independently within the range of 30 μm to 150 μm, while the height *h* = 200 μm remained constant, maintaining a unit silicon substrate period of $P_{x}\;$ = $\;P_{y}$ = 160 μm, supported by a 2 mm-thick high-resistivity silicon substrate. The target operating frequency was set to 0.9 THz. The silicon pillar serves as the basic functional meta-atom of the proposed polarization-multiplexed metasurface. Its rectangular cross-section introduces anisotropic electromagnetic responses for *x*- and *y*-polarized incident waves, allowing the two orthogonal polarization components to experience different effective optical path lengths within the same unit cell. Therefore, by independently tuning the lateral dimensions of the pillar, different phase shifts can be introduced for *x*- and *y*-polarized waves. This unit-cell design forms the physical foundation of the phase and transmission database in Figure 2 and enables the subsequent realization of polarization-dependent focusing. Figure 2b,c correspond to the phase $\varphi_{x}$ and transmission amplitude |$t_{x}$| for *x*-polarization, while Figure 2d,e correspond to the phase $\varphi_{y}$ and transmission amplitude |$t_{y}$| for *y*-polarized incidence, collectively forming the core design database. Here, the transmission amplitude is defined as the magnitude of the electric-field transmission coefficient, $\left|t|=|E_{t}/E_{i}\right|$, where $E_{i}$ and $E_{t}$ are the incident and transmitted electric-field amplitudes, respectively. This definition is applied separately to *x*- and *y*-polarized incidence to obtain $\left|t_{x}\right|$ and $\left|t_{y}\right|$. It should be noted that this quantity represents an electric-field amplitude ratio rather than the transmitted power efficiency.

In the simulations, the plane wave is incident from the silicon substrate side and transmitted through the silicon pillar structure into air. A transmission amplitude larger than unity does not indicate energy gain. For transmission from silicon to air, the Fresnel electric-field transmission coefficient at normal incidence can be written as

(4) $$t=\frac{E_{t}}{E_{i}}=\frac{2n_{s}}{n_{s}+n_{air}}$$

which can be larger than unity because $n_{s}$ > $n_{air}$. However, the corresponding power transmittance should include the refractive-index normalization,

(5) $$T=(n_{air}/n_{s}){\left|t\right|}^{2}$$

or be evaluated from the ratio of transmitted to incident power flux. Therefore, the transmitted power remains physically consistent with energy conservation. Due to the rectangular symmetry of the silicon pillar, the *x*- and *y*-polarized responses are interchanged when the two in-plane dimensions are exchanged. Thus, $\varphi_{x}$($l_{x}$, $l_{y}$) ≈ $\varphi_{y}$($l_{y}$, $l_{x}$) and $t_{x}$($l_{x}$, $l_{y}$) ≈ $t_{y}$($l_{y}$, $l_{x}$). This means that the symmetry is reflected in the correspondence between the *x*- and *y*-polarized response maps, rather than in the self-symmetry of each individual map about $l_{x}$ = $l_{y}$. Crucially, by appropriately selecting ($l_{x}$, $l_{y}$) combinations from this database, it is possible to achieve nearly arbitrary phase shift pairs ($\varphi_{x}$, $\varphi_{y}$) within the range of 0 to 2π in a single silicon pillar while maintaining high optical transmission efficiency. Thus, the main results of independent dual-polarization focusing and high transmission efficiency are directly determined by the controllable anisotropic response of the silicon pillar meta-atoms.

In the proposed design, polarization is employed as the multiplexing degree of freedom. The *x*- and *y*-polarized incident THz waves are regarded as two independent input channels, and each channel is assigned a different phase profile. Therefore, the present metasurface realizes two-channel linear-polarization multiplexing, corresponding to two independently encoded wavefront functions. In this work, the number of independently multiplexed channels is two, corresponding to the two orthogonal linear polarization states.

**Figure 3:**
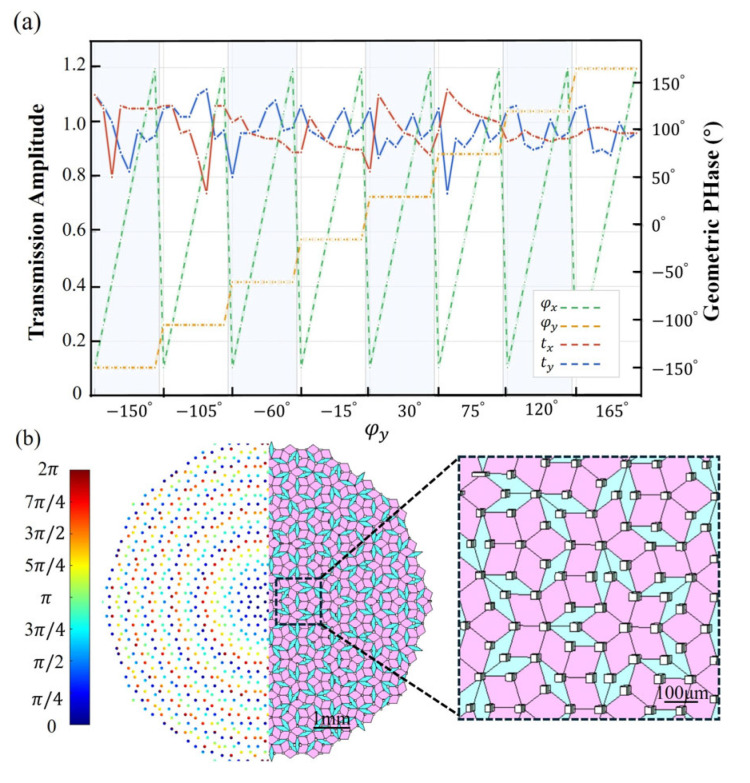
Schematic diagram of metasurface structure design and performance verification. (**a**) 64 sets of structures corresponding to *x-* and *y*-polarizations covering 2π phase and as consistent as possible amplitudes $t_{x}$ and $t_{y}$. (**b**) Phase profile of the designed all-dielectric lens and corresponding metasurface arrangement. The white squares represent the selected silicon pillar meta-atoms placed at the quasicrystal lattice sites, while the magenta and blue rhombi indicate the underlying quasicrystalline tiling.

Figure 3a shows the selected 64 silicon pillar structures used for the polarization-multiplexed metalens. These structures provide eight discrete phase levels for each polarization channel, enabling independent phase control for *x*- and *y*-polarized incidence. During selection, the transmission amplitudes of the two channels were kept as uniform as possible and as close to unity as possible to reduce amplitude-induced focusing distortion. The curves in Figure 3a have been labeled to distinguish the phase and transmission-amplitude responses of the selected structures for the two polarizations. The fivefold quasicrystalline arrangement was selected because it provides an ordered but non-periodic aperture that is suitable for mapping independent phase profiles for the two orthogonal polarization channels. Unlike conventional periodic lattices, the fivefold Penrose-type tiling has long-range order without translational symmetry, which avoids strict periodic sampling constraints in the metasurface aperture. In addition, its 72° rotational symmetry provides a directionally balanced distribution of lattice sites, which is useful for constructing a compact polarization-multiplexed THz focusing device.

Based on the selected meta-atom library, the required phase profiles for the two polarization channels were mapped onto the five-fold rotationally symmetric quasicrystalline lattice. Figure 3b shows the designed phase distribution and the corresponding metasurface arrangement. The white squares represent the selected silicon pillar meta-atoms at the quasicrystal lattice sites, while the magenta and blue rhombi indicate the underlying quasicrystalline tiling. Full-wave electromagnetic simulations at 0.9 THz were then used to evaluate the focusing performance and design flexibility of the lens.

## 3. Simulation Results and Performance Evaluation

**Figure 4:**
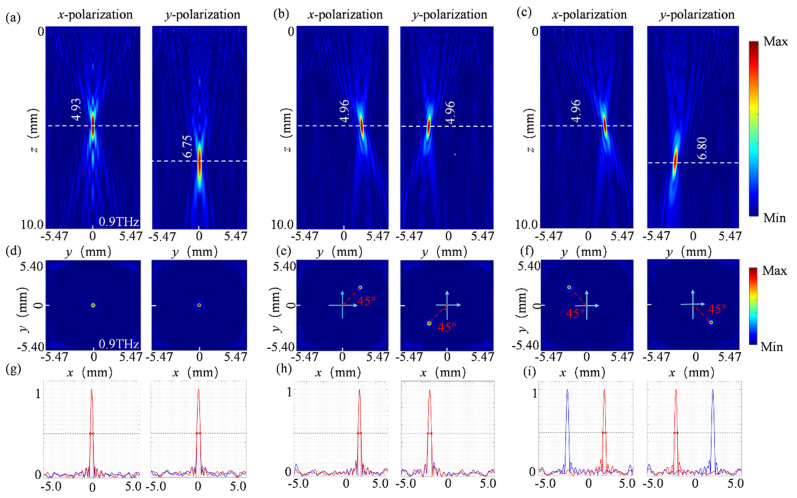
Full-wave electromagnetic simulation verification of the dual-focus metalens performance for polarization-multiplexed quasicrystalline metasurfaces. Simulated normalized electric-field intensity (${\left|E\right|}^{2}$) distributions are shown for three representative designs. White dashed lines mark the designed focal positions. (**a**) For *x-* and *y*-polarized incidence, the two foci are located on the optical axis with focal lengths of 5 mm and 7 mm, respectively. (**b**) The foci of *x*- and *y*-polarized lights are designed to be off-axis, located in the first and third quadrants, respectively, both with a focal length of 5 mm. (**c**) The foci of *x*- and *y*-polarized lights are designed to be located in the second and fourth quadrants, respectively, with focal lengths of 5 mm and 7 mm. (**d**–**f**) The corresponding simulated electric field intensity distributions on their respective focal planes (*x–y* planes), clearly demonstrating the transverse positions and shapes of the focal spots. (**g**–**i**) Cross-sectional profiles along the horizontal (blue solid lines) and vertical (red dashed lines) directions through the center of the focal spots in (**d**–**f**). All simulations were performed at 0.9 THz frequency.

Figure 4 summarizes the simulation results under different complex wavefront configurations, and key performance indicators, such as focal length, spot size, and crosstalk between polarization channels, were accurately extracted through post-processing. Figure 4a,d,g display the simulation results for the first set of configurations, aimed at verifying the lens’s fundamental capability to achieve axial differentiated focusing for orthogonal linearly polarized light. In this design, the theoretical focal length for *x*-polarized light is set to 5.0 mm, while for y-polarized light, it is set to 7.0 mm. The normalized electric field intensity distribution in the *y*–*z* plane shown in Figure 4a clearly indicates that the two energy-concentrated focal points are located at axial positions of 4.93 mm and 6.75 mm, respectively, which closely aligns with the design objectives. The corresponding field distribution in the *x*–*y* plane, shown in Figure 4d, further reveals the transverse morphology of the two focal points, both exhibiting regular circular spots. Figure 4g presents the electric field intensity profile along the horizontal direction at the center of the focal spot, with the full width at half maximum (FWHM) extracted through Gaussian fitting being 0.324 mm and 0.309 mm (for *x*-polarization) and 0.398 mm and 0.405 mm (for *y*-polarization). 

To demonstrate more complex wavefront-manipulation capabilities, Figure 4b,c,e,f,h,i present two advanced configurations for the off-axis spatial arrangement of dual focal points. In the second configuration, shown in Figure 4b,e,h, the focal points of the *x-* and *y-*polarized light are designed to be located in the first and third quadrants, respectively, with a focal length set at 5.0 mm. The *y*–*z* plane field distribution in Figure 4b shows that the two focal points converge axially at the same depth while being completely separated laterally. The two focal points are positioned at axial locations of 4.96 mm and 4.96 mm, and the *x–y* focal plane image in Figure 4e visually illustrates the symmetrical distribution of the two focal points about the optical axis. The intensity profile extracted in Figure 4h shows FWHM values of 0.354 mm, 0.335 mm, 0.343 mm, and 0.349 mm, confirming that the two off-axis focal points maintain good formation while their sizes are effectively controlled, with crosstalk being negligible. The third configuration in Figure 4c,f,i further integrates axial and lateral joint control, placing the focal point of *x*-polarized light with a focal length of 5.0 mm in the second quadrant, while the focal point of *y*-polarized light with a focal length of 7.0 mm is positioned in the fourth quadrant. The simulation results in Figure 4c successfully demonstrate two completely separated focal points in three-dimensional space: one slightly above, at a distance of 4.96 mm, and the other further down, at a distance of 6.80 mm. The focal spot morphology in Figure 4f and the intensity profile in Figure 4i reveal extracted FWHM values of 0.355 mm and 0.337 mm, and 0.410 mm and 0.428 mm, respectively.

The slight variations in focal spot size across all configurations primarily result from changes in numerical aperture and aberrations introduced by off-axis alignment, further validating the precision of the focal position and the quality of the imaging. The differences in focal spot diameter can be explained by the relationship D ∝ λf/L (where λ is the wavelength, *f* is the focal length, and L is the diameter of the incident THz beam), indicating that lenses with shorter focal lengths produce smaller focal spots. These three sets of simulation results robustly demonstrate that the quasicrystal metasurface design method proposed in this study possesses remarkable flexibility in wavefront manipulation. It not only allows for the independent allocation of arbitrarily designed axial focal lengths for two orthogonal polarization states but also enables precise placement of their focal points in any designated quadrant of the transverse plane, achieving complete independent control over the three-dimensional position of the focal point (*x*, *y*, *z*). Notably, the proposed metalens exhibits fast-lens behavior, as its focal length is approximately half of the aperture size, resulting in a relatively high numerical aperture and strong focusing performance. Gaussian characteristics of the focal spots and extremely low crosstalk between polarization channels were observed in all configurations, marking significant application potential for this device in integrated multi-focal imaging, polarization multidimensional information processing, and compact light-field control systems.

**Figure 5:**
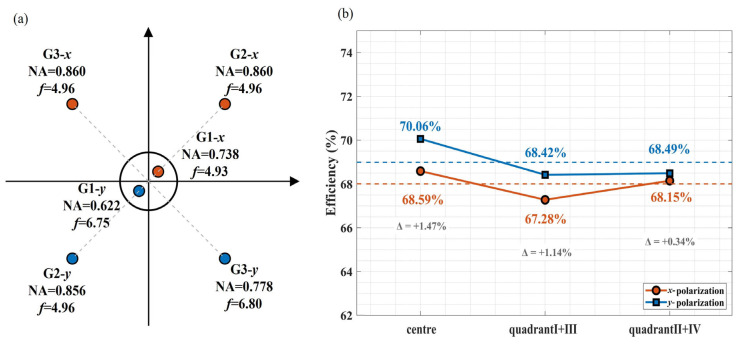
Numerical aperture and efficiency analysis of the quasicrystal bifocal lens at the focal plane. (**a**) Schematic diagram of mode matching region division at the focal plane: Based on the designed phase distribution, the focal plane is divided into four quadrants (I–IV), with labeled design parameter sets (e.g., G1-*x*, G2-*y*) optimized for *x*- and *y*-polarized light in each quadrant, along with the numerical aperture (NA) and focal length (f) extracted from the simulated focal-field distributions in Figure 4. (**b**) Comparison of simulated efficiency under different receiving regions: The efficiencies for *x*-polarized (orange) and *y*-polarized (blue) light are shown when receiving the entire central focal spot region and two specific quadrant combinations (I+III and II+IV). The orange and blue dashed lines represent the average simulated efficiencies of the *x*- and *y*-polarized channels, respectively, over the three receiving regions.

**Figure 6:**
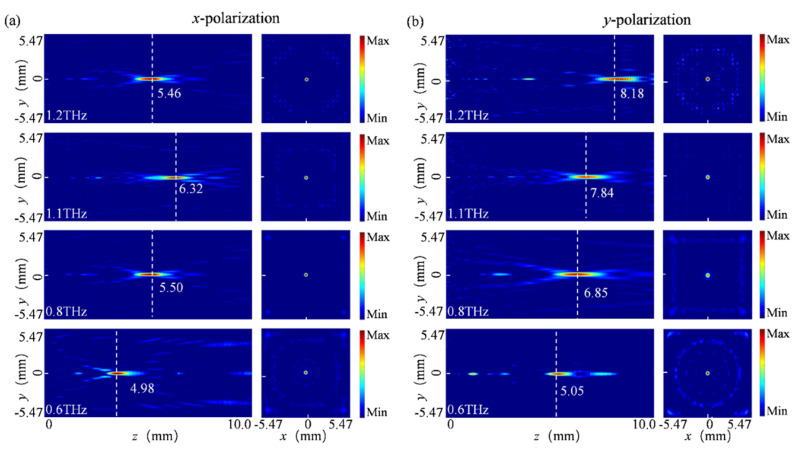
Broadband focusing characteristics of the quasicrystal bifocal lens with central focal lengths (5 mm and 7 mm). (**a**) Simulation results of *x*-polarization covering the broadband *y*–*z* plane and *x*–*y* plane in the 0.6–1.2 THz frequency range. (**b**) Simulation results of *y*-polarization covering the broadband *y*–*z* plane and *x*–*y* plane in the 0.6–1.2 THz frequency range.

**Figure 7:**
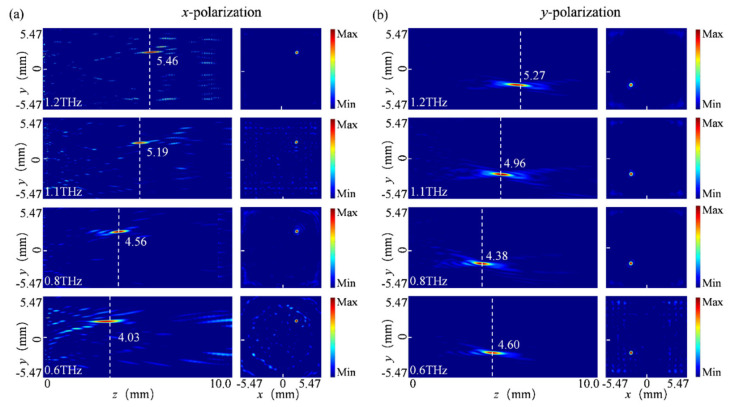
Broadband focusing characteristics of the quasicrystal bifocal lens with focal lengths (5 mm and 5 mm) in the first and third quadrants. (**a**) Simulation results of *x*-polarization covering the broadband *y*–*z* plane and *x*–*y* plane in the 0.6–1.2 THz frequency range. (**b**) Simulation results of *y*-polarization covering the broadband *y*–*z* plane and *x*–*y* plane in the 0.6–1.2 THz frequency range.

**Figure 8:**
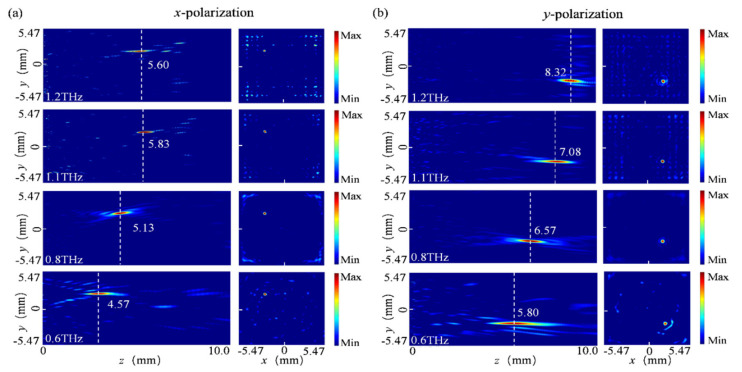
Broadband focusing characteristics of the bifocal quasicrystal lens with focal lengths in the second and fourth quadrants (5 mm and 7 mm). (**a**) Simulation results of *x*-polarization covering the 0.6–1.2 THz frequency band in both the *y*–*z* plane and *x*–*y* plane. (**b**) Simulation results of *y*-polarization covering the 0.6–1.2 THz frequency band in both the *y*–*z* plane and *x*–*y* plane.

From the perspective of system applications, we evaluated the performance of the designed quasicrystal DFL and explored in depth the polarization spatial characteristics of its output light field. We conducted numerical aperture and efficiency simulation studies on the focal plane light field, simulating the potential integration of the lens’s focal light field with subsequent optical systems such as optical fibers and detectors. The numerical aperture (NA) is an important indicator for assessing the divergence angle and mode purity of the light field. Figure 5a illustrates a schematic of the spatial mode matching model used for this analysis. We divided the focal plane into four characteristic regions based on the designed phase distribution and the receiving characteristics of the target optical system. Each region corresponds to a set of optimal unit structure parameters, labeled as G1-*x*,*y* (focal point at the center *x*,*y* polarization), G2-*x*,*y* (focal point in the first and third quadrants), G3-*x*,*y* (focal point in the second and fourth quadrants), as well as the numerical apertures extracted from the simulated focal-field distributions for *x-*polarization (0.738, 0.860, 0.860) and *y*-polarization (0.712, 0.856, 0.778), which also includes the focal lengths designed for each quadrant. This indicates that light of different polarization states is designed to focus on different spatial locations of the focal plane, thereby effectively matching with different modes of the subsequent system. The broadband focusing characteristics of the three representative focal configurations are further analyzed in Figure 6, Figure 7 and Figure 8.

To characterize the efficiency of the proposed metasurface, numerical simulations were conducted using a Gaussian beam with a radius of 5500 μm and a frequency of 0.9 THz, which was incident from the silicon substrate side and transmitted into air. The transmission efficiency of the single structural interface was calculated as η=$I_{out}$/$I_{in}$, where $\;I_{out}$ = ${cn}_{air}\Sigma {\left|E_{out}\right|}^{2}∕4\pi$ and $I_{in}$ = ${cn}_{si}\Sigma {\left|E_{in}\right|}^{2}∕4\pi$ represent the integrated intensity of the transmitted THz beam on the output plane in air and that of the incident THz beam on the reference plane inside the silicon substrate, respectively. Here, $E_{out}$ and $E_{in}$ denote the corresponding electric-field distributions on the air-side output plane and the silicon-substrate-side input reference plane, respectively; c is the speed of light, and $n_{air}$ and $n_{si}$ are the refractive indices of air and silicon, respectively. The results are shown in Figure 5b. When receiving the entire focal region at the center, the efficiencies for *x-* and *y-*polarized light can reach 68.59% and 70.06%, respectively. The efficiency values at the focus located in the first and third quadrants are 67.28% and 68.42%, while the efficiencies in the second and fourth quadrants are 68.49% and 68.15%, respectively.

The proposed polarization-multiplexed quasicrystalline metasurface has several advantages: compact, planar integration; low-loss, all-dielectric operation in the THz regime; independent dual-channel focusing under orthogonal linear polarizations; and flexible three-dimensional focal-position control. The quasicrystalline arrangement also provides non-periodic aperture sampling with rotational symmetry, which helps reduce artifacts associated with strictly periodic lattices. Nevertheless, the current device is passive, and its focusing functions are fixed after fabrication. The performance may also be affected by fabrication tolerances in the silicon pillar dimensions, and experimental validation is still needed. Future work may therefore explore tunable materials, active modulation mechanisms, and fabrication optimization for dynamically reconfigurable polarization-multiplexed THz metasurfaces.

Compared with the recent all-silicon linear-polarization-multiplexed THz metasurface zone plate, which uses a periodic zone-plate-type design with four basic unit cells and a small set of phase states, the present work follows a different route [49]. Here, polarization multiplexing is combined with a five-fold quasicrystalline aperiodic arrangement. A phase and transmission database is built by sweeping the dimensions of rectangular silicon pillars, and 64 meta-atoms are selected to provide eight discrete phase levels for each polarization channel. This multi-level phase strategy offers greater flexibility for mapping polarization-dependent phase profiles onto a deterministic aperiodic lattice. The main distinction of the proposed device is therefore the integration of all-dielectric polarization multiplexing, quasicrystalline spatial arrangement, compact high-NA focusing, and robustness analysis in the THz regime.

## 4. Robustness and Scalability Assessment

To comprehensively evaluate the operational performance of the quasicrystalline DFL designed for the system across a wide frequency band, we conducted full-wave electromagnetic simulations for three representative focal length configurations. These configurations include the center coaxial dual focal lengths, the co-focal lengths in the first and third quadrants, and the off-axis varying focal lengths in the second and fourth quadrants. We analyzed the focusing characteristics variations of (a) *x*-polarization and (b) *y*-polarization within the frequency range of 0.6 THz to 1.2 THz. Based on the simulation results shown in Figure 6, Figure 7 and Figure 8, it is evident that the lens effectively maintains its designed functionality across the wide frequency band of 0.6–1.2 THz. At the target frequency of 0.9 THz, the electric field intensity distribution at the focal plane for all configurations exhibits regular and concentrated bright spots, with the focal point positions aligning closely with design expectations.

As the frequency gradually deviates from the center value, moving towards 0.6 THz or 1.2 THz, a slight diffusion of the focus begins to occur; however, the focusing morphology remains clear at the edges of the band. Nonetheless, once the frequency exceeds this range (below 0.6 THz or above 1.2 THz), the focus rapidly expands, resulting in a significant decrease in the focusing effect, which may ultimately lead to its complete disappearance.

**Figure 9:**
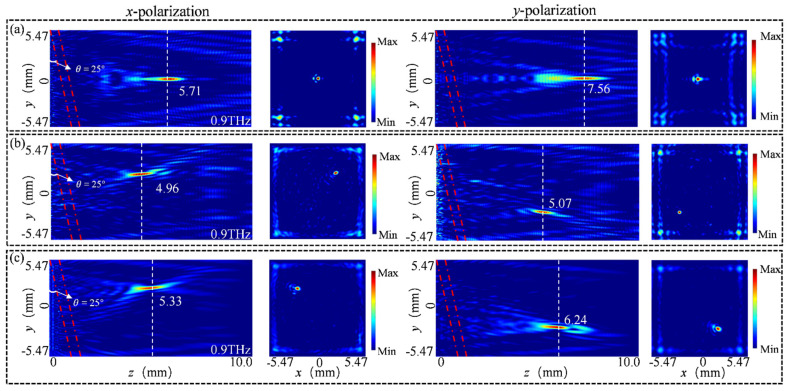
The schematic diagram of testing the anti-interference performance of the quasicrystal bifocal lens. (**a**–**c**) show the *y–z* and *x–y* plane focusing patterns of the metasurface lens tilted at 25° with focal points at: (**a**) center, (**b**) first and third quadrants, and (**c**) second and fourth quadrants, respectively.

To further assess the robustness of the designed quasicrystal double-focus lens in practical application scenarios, we examined its performance under non-vertical incidence conditions, specifically its ability to resist angular interference. Due to software limitations, the incident angle of the plane wave cannot be directly adjusted. We employed an equivalent modeling approach: the plane wave is always incident vertically along the negative *z*-axis, while the equivalent oblique incidence of the beam is achieved by rotating the entire quasicrystal sub-surface structure. This analysis quantitatively investigates the beam fidelity and intensity stability when the structure deviates from the ideal incident conditions. Since the focal point nearly disappears at an inclination angle of 30°, we only consider an angle of 25° for this study. Figure 9a–c present the simulation results of the lens output optical field when the metasurface is tilted at 25° under the target operating frequency of 0.9 THz. By analyzing the electric field intensity distribution ${\left|E\right|}^{2}$ under different observation planes or conditions, we can systematically evaluate the stability of the device’s wavefront-manipulation capabilities at large angle incidences. Even with a 25° oblique incidence, the double-focus structure remains clearly maintained.

**Figure 10:**
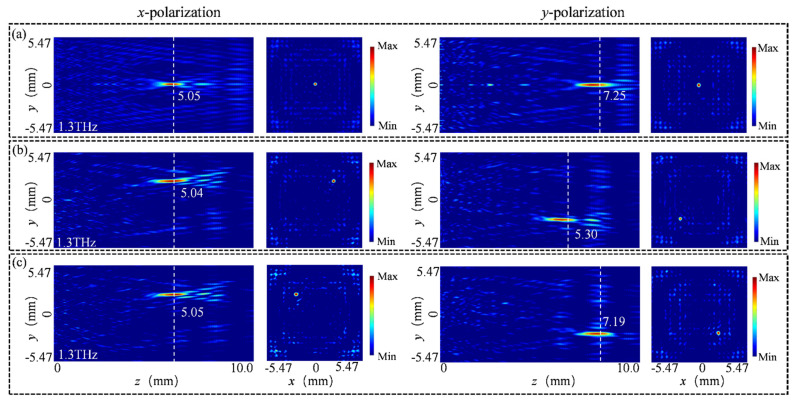
Simulated focusing performance of the redesigned quasicrystalline bifocal metalens at 1.3 THz. (**a**–**c**) Cross-sectional field profiles for three dual-focus configurations: (**a**) central axial foci for *x*- and *y*-polarized incidence, (**b**) off-axis foci in the first and third quadrants, and (**c**) off-axis foci in the second and fourth quadrants.

To validate the reconfigurability and design flexibility of the proposed quasicrystal DFL design method at different frequencies, we reproduced and evaluated the lens performance at 1.3 THz, which exceeds the previously verified broadband range (0.6–1.2 THz). The primary reason for selecting this frequency is that, in the lower THz band, the phase variation range of the unit structure significantly narrows with the increase in wavelength, making it difficult to cover the complete phase range from 0 to 2π, and thus unable to meet the design requirements for 64 sets of independent phase size combinations. However, at the higher frequency of 1.3 THz, despite the shortened wavelength, effective wavefront manipulation can still be achieved by re-optimizing the unit sizes and constructing a phase-size database suitable for this frequency. Figure 10 shows the simulation results of focusing performance for three dual-focus configurations (center coaxial, off-axis in the first and third quadrants, and off-axis in the second and fourth quadrants) based on the redesigned unit-size database at 1.3 THz. 

These results further verify the feasibility of the quasicrystalline design method at a different operating frequency and demonstrate its adaptability through database reconstruction. Beyond the present demonstration, polarization multiplexing may also provide opportunities for optical information encoding and secure communication. Recent work on BB84 quantum-protocol-inspired coherent-state encryption has shown that polarization-related optical states can be used for classical optical encryption and information encoding [50]. From this perspective, polarization-multiplexed THz metasurfaces could be further developed for secure beam routing, polarization-selective information processing, and multifunctional THz communication systems. Future work could combine polarization multiplexing with other degrees of freedom or tunable materials to realize higher-capacity and actively controllable THz metasurface devices.

## 5. Conclusions

This work proposes and systematically investigates an all-dielectric THz metasurface based on a fivefold rotationally symmetric quasicrystalline aperiodic arrangement. By seamlessly integrating polarization multiplexing with the continuous diffraction characteristics of quasicrystals, the proposed device achieves independent dual-focus control within a single platform. The design employs high-resistivity silicon anisotropic pillars as low-loss phase-modulating elements, which directly tailor the propagation phase to encode independent focusing wavefronts for two orthogonal linear polarizations, enabling precise three-dimensional positioning of the dual foci. Full-wave simulation results demonstrate that the metalens achieves a maximum focusing efficiency of 70% at the design frequency of 0.9 THz, while maintaining stable performance over a broad bandwidth from 0.6 to 1.2 THz. Compared with periodic metasurfaces, the continuous diffraction spectrum afforded by the quasicrystalline aperiodic arrangement helps reduce grating lobes and higher-order diffraction, significantly broadening the operational bandwidth and enhancing immunity to interference. Furthermore, the structure exhibits excellent robustness against variations in incident angle and frequency detuning. This study confirms that the combination of quasicrystalline arrangements and all-dielectric polarization multiplexing offers a new technical pathway toward highly efficient, highly integrated, and environmentally robust THz multifunctional planar devices.

## Data Availability

The original contributions presented in this study are included in the article. Further inquiries can be directed to the corresponding authors.
